# Gross Fixed Capital Formation in the Euro Area During the COVID-19 Pandemic

**DOI:** 10.1007/s10272-022-1058-1

**Published:** 2022-08-06

**Authors:** Mirko Licchetta, Eric Meyermans

**Affiliations:** Directorate-General for Economic and Financial Affairs, European Commission, Rue de la Loi 170/Wetstraat 170, 1049 Brussels, Belgium

**Keywords:** E22, E50, E60, H54

## Abstract

This paper examines the impact of the COVID-19 pandemic on gross fixed capital formation across the euro area. The empirical analysis suggests that the intensity of the lockdown measures to contain the spread of the virus and the country-specific structure of the economy along with other traditional drivers, in particular falling output, can explain a large part of the contraction. The bold policy response at the national and EU level mitigated the impact of COVID-19 and supported the recovery. The faster-than-expected rebound in economic activity suggests that the negative economic impact of the pandemic will be more contained than initially feared. However, uncertainty over future health developments remains high, especially given the risks of new more transmissible variants.

Following the outbreak of the COVID-19 pandemic, gross fixed capital formation (GFCF) in the euro area fell very rapidly in the first and second quarter of 2020, much faster than at the height of the global financial crisis. The sharp contraction in GFCF prompted many commentators to highlight the risks that the pandemic could lead to another period of subdued investment growth similar to the one following the global financial crisis, when it took about ten years[1] to return to its pre-crisis level.[2]

However, GFCF recovered (although only partially) at a much faster pace than in the wake of the financial crisis (Figure 1). The multifaceted and sizable policy response at the national and EU level mitigated the impact of the crisis and the plunge in GFCF at the onset turned out to be short-lived. Investment bounced back forcefully in the context of very strong (and temporarily held back) demand and favourable financing conditions (European Commission, 2021a, 2021c). Public investment also picked up considerably.

This paper examines how the COVID-19 pandemic affected investment across the euro area. First, unlike previous investigations that have tended to focus on the impact of the COVID-19 crisis on overall GDP, it assesses the impact of the crisis and lockdown measures on GFCF. Second, this paper estimates the sensitivity of GFCF to lockdown measures over time and across countries supporting the idea of ongoing learning from experiences and gradual adaptation, which includes greater digitalisation. Third, it provides an assessment of the upside and downside risks for GFCF from COVID-19.

## Gross fixed capital formation during the COVID-19 pandemic

Following the COVID-19 shock, gross fixed capital formation contracted by around 23% between the fourth quarter of 2019 and the second quarter of 2020. Over the same period, GDP fell by 15% and the decline in investment was the second largest cause for this overall contraction (following the drop in consumption). This contraction was much larger than the one recorded following the outbreak of the global financial crisis (Figure 2).[3] What was extraordinary about the decline in 2020 was that it all happened

1 In the national accounts (ESA, 2010), gross fixed capital formation covers machinery, equipment, buildings and structures, as well as cultivated biological resources and intellectual property products.

2 After the global financial crisis, the loss of capital stock was the main drag on potential output growth (ECB, 2020).

3 GDP in the first quarter of 2009 declined by around 5.5% relative to the first quarter of 2008, whereas investment fell by around 11%.

* This paper represents the authors' views and not necessarily those of the European Commission.

**Mirko Licchetta**, European Commission, Brussels, Belgium.

**Eric Meyermans**, European Commission, Brussels, Belgium.

Figure 1

**Gross fixed capital formation and GDP in the euro area**

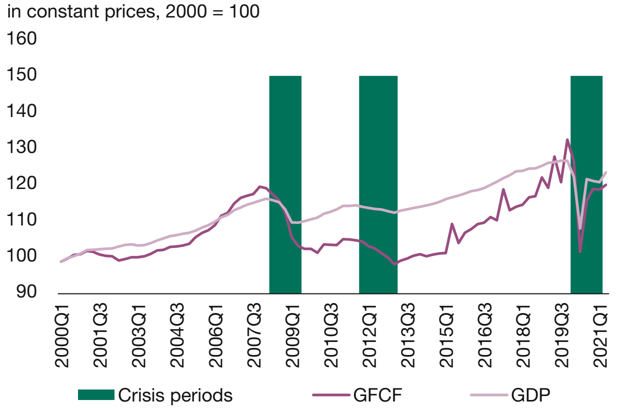

Source: Eurostat.

Figure 2

**Gross fixed capital formation in the euro area during COVID-19 and global financial crisis**

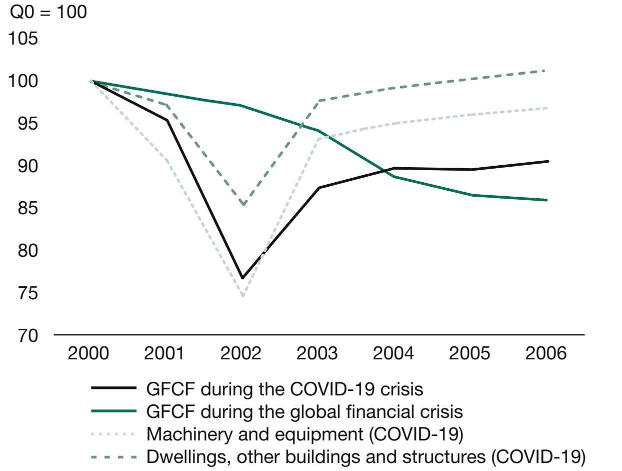

Note: Q0 = 0 is 2008Q1 = 100 for the global financial crisis and 2019Q4 = 100 for the COVID-19 crisis; Q6 = 2009Q3 for the GFC and 2021Q2 for the COVID-19 crisis. Real terms.

Source: Eurostat.

in just two quarters – mainly due to the tightening of lockdown measures to contain the spread of the pandemic (see below).

Lower investments in machinery and equipment (excluding the very volatile intellectual property products data) accounted for most of the fall in the second quarter of 2021 (Figure 2), but it rebounded strongly in the third quarter of 2020. By contrast, dwellings and other buildings and structures contributed less to the contraction and they had recovered their pre-crisis levels by the first quarter of 2021. Investment in intangibles, such as research and development,[4] fell less than investment in machinery and equipment.

At the institutional sector level, the fall in private investment was partly compensated by a symmetric rise in public investment as euro area governments pledged substantial public investment to support the recovery from the pandemic. This was in stark contrast to the period following the global financial crisis (Figure 3), which saw euro area governments cutting back on public investment with the aim of hastening the consolidation of public finances.

The depth of the decline in GFCF between the fourth quarter of 2019 and the second quarter of 2020 varied widely within the euro area, ranging from just below 1% in Finland to 80% in Ireland (Figure 4). Intellectual property – one key and growing component of GFCF – has been particularly volatile in Ireland, Estonia, Cyprus and Luxembourg (see the right-hand side in Figure 4).

Part of these cross-country differences in investment growth can be attributed to differences in the intensity of the lockdown measures (the second quarter of 2020 in Figure 5). As restrictions on movement were lifted between the end of the second and the third quarter of 2020, GFCF rebounded in that third quarter. Lockdown measures were tightened again in the fourth quarter of 2020 on the back of renewed pressures on the member states' health systems; the economic impact of the sec-

Figure 3

**Public and private investment**

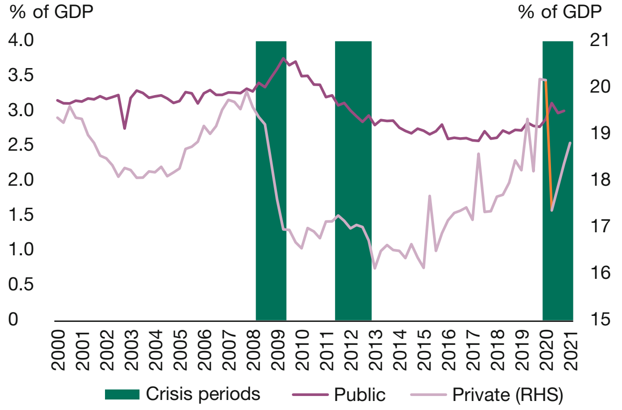

Note: Q0 = 0 is 2008Q1 = 100 for the global financial crisis and 2019Q4 = 100 for the COVID-19 crisis; Q6 = 2009Q3 for the GFC and 2021Q2 for the COVID-19 crisis. Real terms.

Source: Eurostat, institutional sector accounts.

4 The volatile Ireland data are excluded.

Figure 4
**Changes in gross fixed capital formation since the onset of COVID-19**

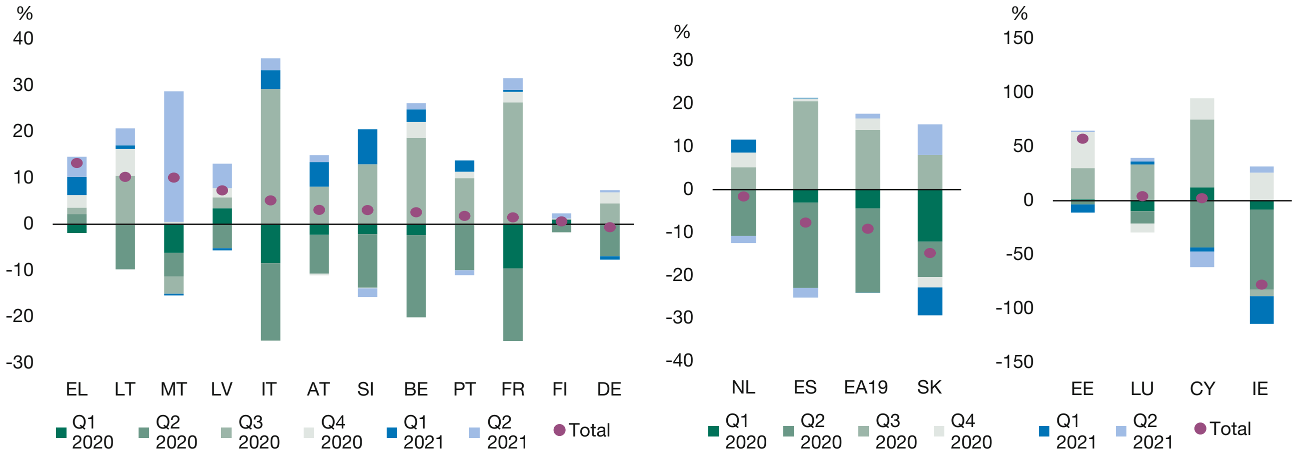

Notes: Data on GFCF for IE, CY, LU and EE show very strong volatility in the intellectual property investment component. Total growth (bullet) measures the compound growth rate (i.e. multiplicative). Given the large size of the growth rates, adding quarterly growth rates (coloured bars) is only a rough approximation of the total growth rate between the first quarter of 2020 and second quarter of 2021.

Source: Eurostat, national accounts.

ond lockdown, however, was more contained than that of the first one.

## COVID-19 related drivers of gross fixed capital formation

The literature suggests a strong negative relationship between governments' lockdown measures and GDP (including its components). This negative impact increases with the intensity of measures (e.g. IMF, 2020; Niermann and Pitterle, 2021), the importance of tourism in the economy and lower quality of governance (e.g. Sapir, 2020). However, over time, economic activity became less sensitive to lockdown measures as firms and households adapted to the new environment (see Figure 5 and the empirical result below).

Figure 5
**Change in gross fixed capital formation and Oxford stringency index in 2020**

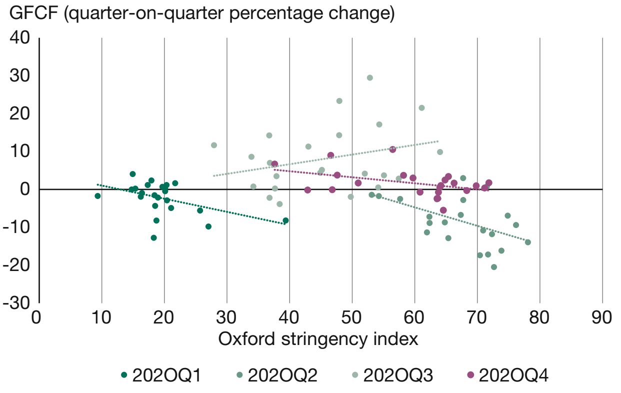

Note: IE, EE, CY and LU excluded from the sample.

Source: Eurostat and Oxford stringency index.

Early evidence suggested that higher uncertainty in the initial phase of the COVID-19 crisis (European Commission, 2021d; Gayer et al., 2021) took a toll on business investment. For example, surveying about 13,500 firms across the EU in 2020, the EIB (2020) reports that about 80% of EU firms considered uncertainty to be an impediment, with some 50% of firms even considering it a major impediment.[5] Gieseck and Rujin (2020) report that heightened uncertainty could have accounted for around one-fifth of the decline in activity by the first half of 2020, with a particularly strong impact on fixed capital formation.

At the beginning of the COVID-19 crisis, financing conditions tightened given the overall uncertainty of the scale and duration of the crisis. However, the increase was short-lived (see Figure 6), following a strong monetary policy response, which prevented financing conditions from tightening in a pro-cyclical way (Lane, 2020). Further financial relief was provided under various state credit guarantee

5 See EIB (2021). European Commission (2020a) reports that firms expected a contraction of 4.5% in capital expenditure in 2020 with more than 40% of participants indicating negative expectations.

Figure 6
**Non-financial corporations cost of credit and composite financial condition indicator**

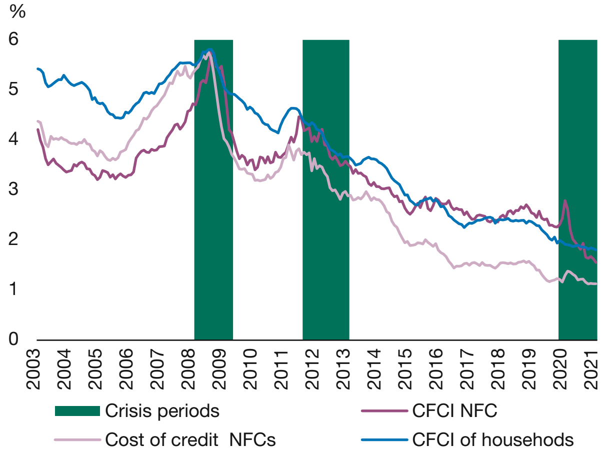

Note: NFC: non-financial corporations; CFCI: composite financial condition indicator.

Source: European Commission.

programmes that supported solvable firms' access to finance for investment (European Commission, 2020b).

Monetary and supervisory authorities supported the financing of investments in several ways. The ECB's monetary policy response mainly consisted of additional asset purchases including via the pandemic emergency purchase programme, ample liquidity provision (mostly via targeted long-term refinancing operations), and easing of collateral standards, while maintaining the deposit facility rate at a record low of -0.5% (since September 2019). At the same time, several national macro-prudential authorities reduced countercyclical capital and systemic risk buffers, while the Single Supervisory Mechanism allowed banks to meet part of their core capital requirements with non-core capital instruments.

Finally, the policy responses at the EU level that supported investment included the mobilisation of all available cash reserves from the European Structural and Investment Funds, putting in place the European instrument for temporary Support to mitigate Unemployment Risks in an Emergency (McDonnell et al., 2021) and the creation of the recovery instrument NextGenerationEU (Alfman et al., 2021).[6] At the national level, the fiscal authorities supported investments via several measures including following the activation of the general escape clause of the Stability and Growth Pact. These measures included emergency spending on health care, short-time work schemes, grants, loan guarantees, loan repayments moratoria, tax deferrals,[7] liquidity support and the roll-out of a vaccination programme.

## Empirical results

The impact of the COVID-19 pandemic on quarterly growth in gross fixed capital formation across the euro area is estimated via a panel error correction model. The model relates investment to output, the past change in capital stock that requires investment to offset capital depreciation,[8] financing costs, a news-based measure of uncertainty[9] and the equity-to-book ratio. To account for the impact of the pandemic, this base model is augmented to include lockdown measures using the Oxford stringency index,[10] a pandemic dummy (equal to 1 for the length of the pandemic since the second quarter of 2020[11]) that captures the net impact of other factors including fiscal and monetary policy responses.[12]

This section reports estimation results for a panel error correction model, covering 15 euro area member states[13] from the first quarter of 2002 to the second quarter of 2021.[14] First, the equilibrium relationship is estimated between the level of gross fixed capital formation (I) and the level of real GDP, the financing cost

6 The Recovery and Resilience Plan's total GDP impact generated during the 2021-2022 period is expected to be approximately 1.2% of the EU's 2019 real GDP, with a noticeable impact on the GFCF for a significant number of member states. See European Commission (2021b).

7 And in some countries the introduction of temporary suspensions of bankruptcy proceedings.

8 Net capital stock data with quarterly frequency are interpolated from AMECO annual capital stock series OKND.

9 Uncertainty is measured by the Economic Policy Uncertainty index based on newspaper articles regarding policy uncertainty. However, part of the impact of rising uncertainty may also be captured by other explanatory variables such as the pandemic dummy and lockdown measures.

10 The Oxford COVID-19 stringency index varies between 1 (very loose) and 100 (very tight). It includes several dimensions: (i) lockdown and closure measures; (ii) economic response and (iii) health system measures (see Halle et al., 2020).

11 Complemented with a dummy for the first quarter of 2020 as the first weeks of this quarter were not yet affected by the pandemic.

12 A dummy equal to 1 for the length of the pandemic since the second quarter of 2020, complemented with a dummy equal to 1 for the first quarter of 2020.

13 IE, EE, CY and LU are not included as they show strong variability in the intellectual property products component.

14 The main data sources are Eurostat National Accounts and Sectoral Accounts, Oxford COVID-19 Government Response Tracker project and AMECO.

Table 1
**Equilibrium (semi-)elasticities**

| | GDP | USER | PB_ ratio | Lock-down | DUM_ COVID | DUM_ GFC |
|---|---|---|---|---|---|---|
| Equilibrium (semi-)elas-ticities | 0.99 | -0.56 | 0.14 | -0.14 | 0.08 | -0.02 |

Note: 2002Q1-2021Q2, including BE, DE, EL, ES, FR, IT, LV, LT, MT, NL, AT, PT, SI, SK and FI.

Source: Authors' calculations.

(USER),[15] the equity to book value ratio (PB_ratio)[16] and a global financial crisis dummy (DUM_GFC). To capture the specific impact of the pandemic, this equilibrium relationship is augmented with the Oxford stringency indicator (LOCKDOWN) and a dummy for the net impact of all other factors affecting investment during the pandemic including a proxy for the monetary and fiscal policy response to the crisis (DUM_COVID).[17] The estimated equation is:

$$(1)\quad \ln\left(I_{,it}\right) = \beta_0 + \beta_1 ln\left(GDP_{it}\right) + \beta_2 USER_{it} + \beta_3 PB\_ratio_{it} + \beta_4 LOCKDOWN_{it} + \beta_5 DUM\_COVID_t + \beta_6 DUM\_GFC_t + ECT_{it}$$

with the subscripts i and t referring to the countries and quarters respectively, and whereby $\beta_1$, $\beta_3 > 0$ while $\beta_2$, $\beta_4$, $\beta_6 < 0$ and the sign of $\beta_5$ is ambiguous as it covers a whole range of transmission channels. ECT is the error correction term used in the second step of the regression analysis. Table 1 shows that the point estimates all have the expected sign. The Pedroni and Kao panel co-integration test suggests that the null hypothesis of no co-integration can be rejected at a high level of confidence.

Next, the short- to medium-term dynamics are estimated with pooled generalised least squares,[18] using instrumental variables,[19] i.e.

$$(2)\quad \Delta\ln\left(I_{it}\right) = \gamma_0 \sum_{j=1}^{4} \gamma_{1j}\, \Delta ln\left(GDP_{it-j}\right) + \gamma_2 \Delta ln\left(Captial_{it-1}\right) + \gamma_3 \Delta USER_{it} + \gamma_4 \Delta PB\_ratio_{it} + \gamma_5 \Delta LOCKDOWN_{it} + \gamma_6 DUM\_COVID_t + \gamma_7 DUM\_GFC_t + \gamma_8 ECT_{it-1} + u_{it}$$

with $\Delta$ the operator comparing one quarter to the previous quarter, and with $\sum_{j=1}^{4} \gamma_{1j} > 1, \gamma_2, \gamma_4 > 0$.

Table 2 reports the main estimation results. Variant V1 is the baseline model capturing the dynamics towards equilibrium. Most point estimates have the expected sign and are statistically significant. Several robustness tests were performed, indicating that the qualitative nature of these results is broadly unchanged if (i) a stricter version of the Oxford indicator that focuses only on mobility restrictions is considered (V1-lockdown), (ii) investment in dwellings is excluded (V1-dwellings), (iii) the error correction term is estimated excluding pandemic related variables (V1-technical),[20] (iv) replacing the change in the lockdown measures by its level did not change the significance of the point estimates, (v) estimation period is limited to the pre-pandemic period (V1-pre 2020), (vi) not enough degrees of freedom are available to obtain stable estimates for some important COVID-19 related factors such as the vaccination rate that took off in the first quarter of 2021.

## Lockdown measures

The econometric results suggest that quarterly growth in GFCF decreases with the tightening of lockdown measures. This statistically significant finding suggests that a ten-point tightening in the Oxford stringency index leads on average to a contraction of about 2.5 percentage points in GFCF quarter on quarter growth (V1 in Table 2).

15 The real user cost of capital is measured as

$$USER_{it} = \frac{IR_{it} + \tau - \left(\frac{PC_{it+1}}{PC_{it}} - 1\right)(1-\tau)}{1 + IR_{it}} \frac{PC_{it}}{P_{it}},$$

with IR measured as the bank lending rate, $\tau$ the rate of capital depreciation, PC the price of capital, and P the price of output. The expected price change is assumed to be equal to the observed past change.

16 The price/book ratio for the Europe STOXX 600 index is taken as a proxy for the Tobin Q.

17 A dummy equal to 1 from the first quarter of 2020 to the second quarter of 2021, and zero during other periods.

18 Allowing for correlation between the random components across member states.

19 Including lagged explanatory variables and the policy variables excluding its cyclical component estimated via the Hodrick-Prescott filter.

20 In all variants, except V2-technical, the error correction term (ECT) for the entire sample is estimated based on an equilibrium equation (1) as reported in Table 1. For V2-technical, the error correction terms are obtained re-estimating equation (1) for a sample ending in the fourth quarter of 2019, and fitting the error correction term from the first quarter of 2020 to second quarter of 2021 using observed explanatory variables and point estimates of the re-estimated equation 1.

Figure 7
**Responsiveness to a change in lockdown measures across the euro area**

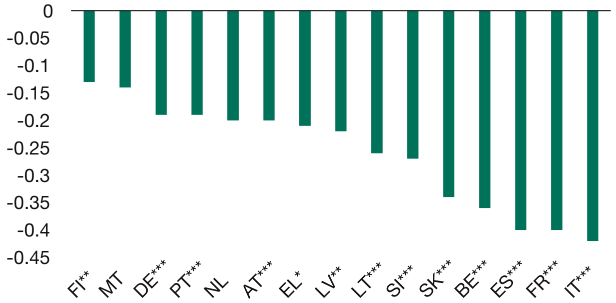

Note: Based on V4 in Table 2. Point estimate significance *** p<0.001, ** p<0.05 and * p<0.

Source: Authors' estimates.

Figure 8
**Sensitivity to a change in lockdown measures and share of contact-intensive sectors in value added**

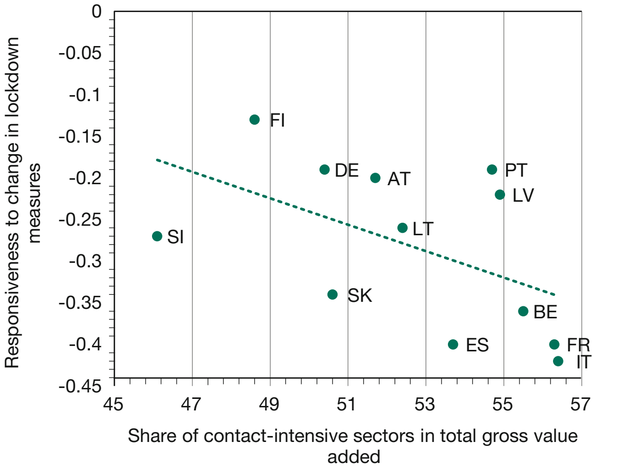

Notes: Contact-intensive sectors refer to wholesale and retail trade, transport, accommodation and food service activities (NACE2 Rev2 classification: G-I); arts, entertainment and recreation (R-U); information and communication (J); financial and insurance activities (K); real estate (L); professional, scientific and technical activities (M); and administrative and support service activities (N). Only member states with 0.05 significance are shown.

Source: Authors' estimates and Eurostat national accounts.

The sensitivity of GFCF to the lockdown measures (V2 in Table 2)[21] decreases over time. This perhaps reflects learning from experiences and gradual adaptation, which includes greater digitalisation. Along these lines, earlier research demonstrates that the impact of the second and third wave on turnover in the various countries was substantially different from that of the first wave, as turnover reductions were relatively subdued in the member states that suffered most in the first wave.

The sensitivity of GFCF also differs across member states. It is the strongest in Italy and the weakest in Malta and Finland (V3 and Figure 7).[22] Such cross-country differences in responsiveness to the lockdown measures might reflect differences in economic structure such as the share of tourism and contact-intensive sectors in the economy (Coutinho et al., 2021). Figure 8 confirms that the responsiveness to the lockdown measures increases with the size of contact-intensive sectors (as a share of total gross value added). In turn, these lockdown measures lowered private consumption and exports, thereby putting additional downward pressure on GDP and consequently also on investment.

## The policy response

The pandemic dummy is found statistically significant (see V1 in Table 2). As such, the dummy captures the role of various factors including the response of monetary and fiscal policy during the COVID-19 crisis. To disentangle GFCF's support of the policy response, the base model (V1) is augmented with a proxy for the monetary and fiscal policy interventions (see V4 in Table 2) while keeping the parameter of the confinement measures constant over time and across member states and keeping a dummy to capture all other COVID-19 related factors.

The change in the ECB balance sheet (as measured by the change in total liabilities during the pandemic) is used as a proxy for the monetary policy related intervention. As for the fiscal policy response, it is measured by general government net lending (as a share of GDP).

The significant positive point estimate for monetary policy suggests that it supported investment through the normalisation of financial market conditions and the provision of credit to the banking sector at favourable rates that helped banks to grant loans to solvable firms.[23] Interestingly, both the monetary policy and financing conditions positively affect GFCF. As the latter reflects mostly market risk premia, the effect of the ECB policy measures are already somehow captured by the financing condition

21 V2 allows the point estimate of the lockdown measures to vary across the six quarters during which the pandemic was hitting the euro area.
22 V3 allows for the point estimate of the lockdown measures to vary across the 15 euro area member states in the sample.

23 Caveat: keeping the coefficients fixed over time and per country may imply that the lower sensitivity of households and firms to lockdown measures during the second phase of the COVID-19 crisis is not captured. As a result, there is a risk of overestimating the impact of the policies.

Figure 9

**Decomposition of the changes in gross fixed capital formation during COVID-19**

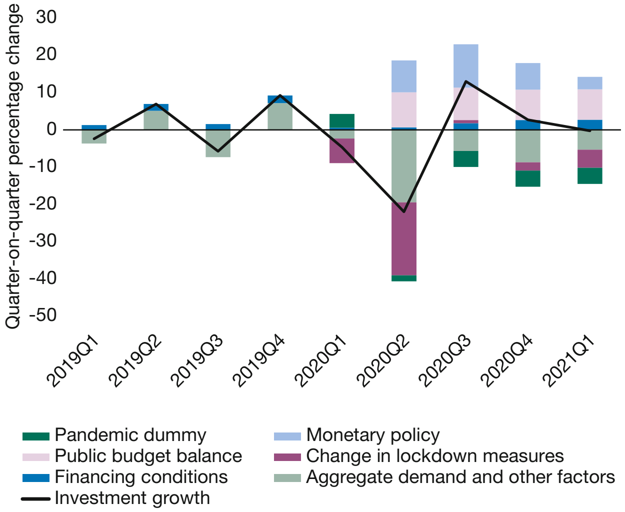

Notes: Model estimation based on variant V4 in Table 2 evaluated for the explanatory variables at EA19 aggregate, i.e. the plotted value is equal to the corresponding point estimate multiplied with the observed change/level of the explanatory variable. Legend: Pandemic dummy refers to the variable DUM_COVID in equation 2; Change in lockdown measures refers to variable LOCKDOWN; Financing condition refers to the sum of variables USER, PB_ratio and UNCERTAINTY; Public budget balance refers to GGNB; Change in ECB liabilities refers to ECB_L.

Source: Authors' estimates.

variable. The presence of an additional, large and positive impact of ECB balance sheet policies on GFCF could reflect confidence-related effects (Schnabel, 2021).

The significant negative point estimate for the public budget balance suggests that the increase in headline deficit supported investment by countering the downward impact of the pandemic shock on aggregate demand (Bellia et al., 2021).[24] Figure 9 provides an overview of the contribution of the various drivers of GFCF during the COVID-19 crisis.

## Long-term impacts of COVID-19

### Upside risks

The pandemic accelerated investment in ICT infrastructure[25] to accommodate the rise in online work and digital sales. The McKinsey Global Institute Report (2021) expects such changes will have the potential to increase annual productivity growth by about one percentage point up to 2024. Also notable, investment in intellectual property products (e.g. investment in software and research and development) held up better than investment in machinery and equipment. This might be because the exchange of intellectual property products involves less physical interaction.

The pandemic also disrupted the functioning of global value chains (GVCs). The fear of a repeat of a pandemic may then strengthen the incentives to bring production closer to home,[26] thus requiring additional investment. But the available evidence on the impact of COVID-19 on GVCs is somewhat ambiguous.[27] At the same time, such reshoring may limit countries' opportunities to exploit their comparative advantages thereby lowering the return on capital and incentives to invest.

### Downside risks

Available evidence suggests that much of the initially feared long-run COVID-19 crisis damage has been avoided thanks to the bold policy response at the national and EU level. However, there remain some risks that might dampen investment going forward, especially in case of a re-intensification of the pandemic (ECB, 2020; IMF, 2021).

If the emergency policy support measures for firms are lifted too abruptly, it might contribute to an increase in corporate distress. This in turn may intensify the financing constraints on investment. For example, OECD (2021) expects insolvencies to increase significantly in the next two years, particularly in high-contact services sectors, admittedly from artificially low levels.

At the same time, the continuation of support policies could carry the risk of locking capital and labour in unproductive sectors, hindering business dynamism over the medium to long term (Claeys et al., 2021; Ebeke et al., 2021). Nevertheless, preliminary evidence suggests that this effect remains modest (Helmersson et al., 2021; Cros et al., 2021), and that business creation has rebounded since the second quarter of 2021 (Eurostat, 2021).

Excessive corporate debt burden accumulated during the pandemic could also act as a drag on investment.[28] For

24 On the combined effect of monetary and fiscal policy following the outbreak of the pandemic.

25 Bellmann et al. (2021) report that almost 30% of the surveyed German companies reported that the pandemic accelerated the introduction of digital technologies.

26 Javorcik (2020) expects that primarily Eastern European and the Southern Mediterranean countries will benefit from "re-shoring" or "near-shoring".

27 The pandemic limited the mobility of goods and persons including managers, but it gave a boost to digitalisation (Simola, 2021).

28 On the accumulation of debt during the COVID-19 crisis in the non-financial corporations and related risks for investment decisions, see ECB (2021).

Table 2

## Point estimates of the panel error correction model

Dependent variable: d ln of investment in constant prices

| | V1 | V1-lockdown | V1-dwellings | V1-technical | V1-pré2020 | V2 | V3 | V4 |
|---|---|---|---|---|---|---|---|---|
| First lag of real GDP growth | 0.40*** | 0.29*** | 0.40*** | 0.55*** | 1.10*** | 0.55*** | 0.38*** | 0.68*** |
| Second lag of real GDP growth | 0.08 | 0.11 | 0.09 | 0.15 | 0.11 | 0.15 | 0.08 | 0.23** |
| Third lag of real GDP growth | 0.17** | 0.22** | 0.19* | 0.23** | 0.39* | 0.33** | 0.16* | 0.44*** |
| Fourth lag of real GDP growth | 0.24* | 0.23* | 0.21 | 0.33** | -0.28 | -0.02 | 0.24* | -0.20 |
| First lag of capital stock growth | 0.03*** | 0.03*** | 0.04*** | 0.03*** | 0.03*** | 0.03*** | 0.03*** | 0.03*** |
| Change in financing cost (USER) | -0.46** | -0.42** | -0.46** | -0.45** | -0.27 | -0.41** | -0.42** | -0.35* |
| GFC dummy | 0.00 | 0.00 | 0.01 | 0.01 | 0.00 | 0.00 | 0.00 | 0.00 |
| Newbased risk index (UNCER) | 0.00** | 0.00** | 0.00** | -0.00*** | 0.00* | 0.00* | 0.00** | 0.00* |
| Change in equity/book ratio (PB_ratio) | 0.05*** | 0.05*** | 0.05*** | 0.03* | 0.05*** | 0.05*** | 0.05*** | 0.05*** |
| Change in ECB liabilities during pandemic (ECB_L) | | | | | | | | 0.55** |
| Public budget balance (% of GDP) during pandemic | | | | | | | | -0.77* |
| Change in lockdown measures (all) (LOCKDOWN) | -0.27*** | | -0.26*** | -0.25*** | | | | -0.34*** |
| Change in lockdown measures 20Q1 (all) | | | | | | -0.56*** | | |
| Change in lockdown measures 20Q2 (all) | | | | | | -0.26*** | | |
| Change in lockdown measures 20Q3 (all) | | | | | | -0.46*** | | |
| Change in lockdown measures 20Q4 (all) | | | | | | -0.26** | | |
| Change in lockdown measures 21Q1 (all) | | | | | | -0.23 | | |
| Change in lockdown measures 21Q2 (all) | | | | | | 0.43** | | |
| Change in lockdown measures (only mobility) | | -0.32*** | | | | | | |
| Pandemic dummy (DUM_COVID) | 0.04*** | 0.05*** | 0.05*** | 0.05*** | | 0.04*** | 0.04*** | -0.03 |
| Pandemic dummy 2020Q1 | 0.02 | 0.02 | 0.02 | 0.02 | | 0.08* | 0.02 | 0.04* |
| Lagged error correction term (ECT) | -0.14*** | -0.15*** | -0.24*** | -0.11*** | -0.12*** | -0.14*** | -0.14*** | -0.13*** |
| Change in lockdown measures (all) - BE | | | | | | | -0.36*** | |
| Change in lockdown measures (all) - DE | | | | | | | -0.19*** | |
| Change in lockdown measures (all) - EL | | | | | | | -0.21* | |
| Change in lockdown measures (all) - ES | | | | | | | -0.40*** | |
| Change in lockdown measures (all) - FR | | | | | | | -0.40*** | |
| Change in lockdown measures (all) - IT | | | | | | | -0.42*** | |
| Change in lockdown measures (all) - LV | | | | | | | -0.22** | |
| Change in lockdown measures (all) - LT | | | | | | | -0.26*** | |
| Change in lockdown measures (all) - MT | | | | | | | -0.14 | |
| Change in lockdown measures (all) - NL | | | | | | | -0.20 | |
| Change in lockdown measures (all) - AT | | | | | | | -0.20*** | |
| Change in lockdown measures (all) - PT | | | | | | | -0.19*** | |
| Change in lockdown measures (all) - SI | | | | | | | -0.27*** | |
| Change in lockdown measures (all) - SK | | | | | | | -0.34*** | |
| Change in lockdown measures (all) - FI | | | | | | | -0.13** | |
| Autocorrection of error term | -0.37*** | -0.36*** | -0.35*** | -0.37*** | -0.41*** | -0.39*** | -0.37*** | -0.40*** |
| Country fixed effects | Yes | Yes | Yes | Yes | Yes | Yes | Yes | Yes |
| | | | | | | | | |
| Adjusted R-squared | 0.29 | 0.30 | 0.30 | 0.27 | 0.31 | 0.32 | 0.30 | 0.33 |
| Number of observations | 1.082 | 1.082 | 1.010 | 1.082 | 992 | 1.082 | 1.082 | 1.059 |
| Number of explanatory variables | 29 | 29 | 28 | 29 | 26 | 34 | 43 | 31 |

Note: 2002Q1-2021Q2, including BE, DE, EL, ES, FR, IT, LV, LT, MT, NL, AT, PT, SI, SK and FI. Natural logarithm changes of one quarter compared to the previous quarter. Net capital stock data with quarterly frequency are interpolated form AMECO annual capital stock series OKND. Pooled generalised least squares; lagged and Hodrick-Prescot filtered series as instrumental variables. Country fixed effects included. *** p<0.001, ** p<0.05 and * p<0.1.

Source: Authors' estimation.

example, non-financial corporations debt-to-GDP ratio (consolidated measure) rose from 77.2% in the first quarter of 2020 to 84.7% in the first quarter of 2021 – of which the largest part seems to be concentrated in a subset of already highly leveraged companies. Such increases in debt might strengthen deleveraging needs thereby discouraging investment.[29]

## Conclusion

This paper suggests that lockdown measures to limit the spread of the virus had a strong adverse impact on gross fixed capital formation across the euro area. The impact varied across countries and over time reflecting partly cross-country differences in economic structure and gradual learning and adaptation by economic agents.

The strong rebound in investment in a context of very strong (and temporarily held back) demand, favourable financing conditions and supportive public investments (European Commission, 2021c) provides reasons for optimism. However, it is still too early to assess the long-term impact of the COVID-19 crisis on GFCF. Available evidence suggests that much of the long-run damage initially feared might have been avoided thanks to the bold policy response at the height of the pandemic and the comprehensive recovery strategy that has ensued.

29 Microsimulations by Bénassy-Quéré et al. (2021) suggest that in France the debt overhang caused by the crisis could reduce corporate investment by almost 2% during the recovery phase. However, the authors do not take into account the impact of the French recovery plan.

## References

[CR1] Alfman E, Engels S, Langedijk S, Pfeiffer P, in’t Veld J (2021). An overview of the economics of the Recovery and Resilience Facility. Quarterly Report on the Euro Area.

[CR2] Bellia M, Calès L, Frattarolo L, Monteiro D, Petracco Giudici M (2021). COVID-19: the stabilising impact of EU bond issuance on sovereigns and banks. Quarterly Review on the Euro Area.

[CR3] Bellmann, L., P. Bourgeon, C. Gathmann, P. Gleiser, C. Kagerl, E. Kleifgen, C. König, U. Leber, D. Marguerit, L. Martin, L. Pohlan, D. Roth, M. Schierholz, J. Stegmaier and A. Aminian (2021, 5 August), The pandemic has boosted firm investments in digital technologies, *VoxEU*.

[CR4] Bénassy-Quéré, A., B. Hadjibeyli and G. Roulleau (2021, 27 April), French firms through the COVID storm: Evidence from firm-level data, *VoxEU*.

[CR5] Claeys, G., M. Hoffmann and G. Wolff (2021, 7 January), Corporate insolvencies during COVID-19: keeping calm before the storm, *Bruegel Blog*.

[CR6] Coutinho L, Vukšić G, Zeugner S (2021). International tourism decline and its impact on external balances in the euro area. Quarterly Report on the Euro Area.

[CR7] Cros, M., A. Eupalard and P. Martin (2021), Will Schumpeter catch COVID-19? Evidence from France, *VoxEU*.

[CR8] Ebeke, C., N. Jovanovic, L. Valderrama and J. Zhou (2021), Corporate Liquidity and Solvency in Europe during COVID-19: The Role of Policies’, *IMF Working Papers*, 21/56.

[CR9] ECB (2020), The scarring effects of past crises on the global economy, *ECB Economic Bulletin*, 8/2020.

[CR10] ECB (2021), Financial Stability Review, May.

[CR11] EIB (2020), The EIB Investment Survey, 2020 EU overview.

[CR12] EIB (2021), Investment Report 2021/2022: Recovery as a springboard for change, Part II Recovery from the COVID-19 pandemic, scarring and asymmetry.

[CR13] European Commission (2020a), EU R&D Survey.

[CR14] European Commission (2020b), Policy measures taken against the spread and impact of the coronavirus — 8 December 2020.

[CR15] European Commission (2021a), Business and Consumer Survey carriedout in April 2021.

[CR16] European Commission (2021b), Spring 2021 Economic Forecast.

[CR17] European Commission (2021c), Autumn 2021 Economic Forecast.

[CR18] European Commission, (2021d), Economic Sentiment and Employment Expectations up in the EU and the euro area.

[CR19] Eurostat (2021), Quarterly registrations of new businesses and declarations of bankruptcies — statistics.

[CR20] Gayer, C., A. Reuter and F. Morice (2021, 29 November), Special topic: new survey-based measure of economic uncertainty, *VoxEU*.

[CR21] Gieseck, A. and S. Rujin (2020), The impact of the recent spike in uncertainty on economic activity in the euro area, *ECB Economic Bulletin*, 6/2020.

[CR22] Halle, T. et al. (2020), A global panel database of pandemic policies (Oxford COVID-19 Government Response Tracker). 10.1038/s41562-021-01079-8 33686204

[CR23] Helmersson, T., L. Mingarelli, B. Mosk, A. Pietsch, B. Ravanetti, T. Shakir and J. Wendelborn (2021), Corporate zombification: post-pandemic risks in the euro area, ECB *Financial Stability Review*, May 2021.

[CR24] IMF (2020), A Long and Difficult Ascent, World Economic Outlook, October.

[CR25] IMF (2021), Austria — Selected Issues, *Staff Country Report*.

[CR26] Javorcik, B. (2020), Global supply chains will not be the same in the post-COVID-19 world, in R. Baldwin and S. Evenett (eds.), *COVID-19 and Trade Policy: Why Turning Inward*, CEPR Press.

[CR27] Lane, P. (2020, 13 March), The Monetary Policy Package: An Analytical Framework, *ECB Blog*.

[CR28] McDonnell C, Boussard J, Justo I, Mohl P, Mourre G, Stovicek K (2021). The SURE instrument — key features and first assessment. Quarterly Report on the Euro Area.

[CR29] McKinsey Global Institute Report (2021), Will productivity and growth return after the COVID-19 crisis?

[CR30] Niermann, L. and I. Pitterle (2021), The COVID-19 crisis: what explains cross country differences in the pandemic’s short-term economic impact?, *MPRA Paper*, 107414

[CR31] OECD (2021), Economic Policy Survey.

[CR32] Sapir, A. (2020), Why has COVID-19 hit different European Union economies so differently, *Bruegel Policy Contribution Issue*, 18.

[CR33] Schnabel, I. (2021), Asset purchases: from crisis to recovery, speech delivered at the Annual Conference of Latvijas Banka on “Sustainable Economy in Times of Change”.

[CR34] Simola, H. (2021), The impact of Covid-19 on global value chain, *BOFIT Policy Brief*, 2/2021, Bank of Finland.

